# Trichoblastomas derived from the facial skin with tactile hair in aged house musk shrews (*Suncus murinus*)

**DOI:** 10.1186/s42826-022-00147-y

**Published:** 2022-12-06

**Authors:** Tohru Kimura

**Affiliations:** Laboratory Animal Science, Joint Faculty of Veterinary Medicine, Yamaguchi University, 1677-1, Yoshida, Yamaguchi, 753-8515 Japan

**Keywords:** Basaloid cells, Benign hair follicle tumors, Free-floating cells, Histopathology, Microcysts, Serum amyloid A, Serum biochemistry, *Suncus murinus*, Tactile hair, Trichoblastomas

## Abstract

**Background:**

Benign hair follicle tumors are relatively rare cutaneous neoplasms arising from hair follicle differentiation. These tumors are slow-growing solitary papules or nodules in the head, face or neck. The aim of this study was to describe 2 cases of trichoblastomas in tactile hair skin incidentally encountered in aged house musk shrews (*Suncus murinus*). In addition, this case report clarifies whether the characteristics in the tactile hair skin of *Suncus murinus* are different from those in humans and other animals.

**Case presentation:**

The animals were investigated the characteristics of the clinical findings, hematological and serum biochemical profiles (particularly, serum amyloid A levels (vSAA)), and histopathological results. *Suncus murinus* with the facial tumor showed weight loss and coarse fur. Hematological examinations indicated microcytic and normochromic anemia. Although few apparent changes were serum biochemically found in *Suncus murinus*, vSAA levels moderately increased and revealed inflammatory reactions. These lesions histopathologically showed the basaloid islands comprising peripheral palisading and dilated microcysts containing variable admixtures of free-floating cells such as neoplasm cells, giant cells, clear cells, mononuclear cells and erythrocytes.

**Conclusions:**

The author concluded that trichoblastomas in *Suncus murinus* revealed growth and morphological characteristics that recapitulate part of embryological development in the tactile hair follicles. In the histological structure, their trichoblastomas in the tactile hair skin were different from those found in humans and animals such as cats, dogs and other wildlife.

## Background

Benign hair follicle tumors are relatively rare cutaneous neoplasms defined by the type and degree of hair follicle differentiation. These tumors generally occur on the head and neck of human adults as a nondescript slow-growing solitary papule or nodule. Tumors of the epidermal appendages have been classified into 4 groups, which exhibit histologic features analogous to those of hair follicles, sebaceous glands, apocrine glands and eccrine glands [1]. In veterinary medicine, benign hair follicle tumors include trichoblastomas, trichoepitheliomas, pilomatricomas, tricholemmomas and trichofolliculomas [2]. To the best of the author’s knowledge, there is, however, no information available about tumors of tactile hair skin in veterinary medical fields.

Laboratory house musk shrews (*Suncus murinus*) belong to the family Soricidae of the order Insectivore. *Suncus murinus* is similar in body length to mice and range in body weight (male: 50–70 g, female: 30–50 g, reference values in the laboratory of Laboratory Animal Science). *Suncus murinus* is mainly insectivorous and this species eat a wide range of invertebrates. *Suncus murinus* is generally solitary and has a high metabolic rate necessitating frequent feeding. The life span of *Suncus murinus* (1.0–1.5 years, reference values in the laboratory of Laboratory Animal Science) is shorter than that of rodent mice (2.0–2.5 years). *Suncus murinus* are not known for having well-developed vision and tactile communication prominently occurs between mates, and between individuals in aggressive encounters.

To date, there is very limited information in the literature regarding neoplasia in *Suncus murinus.* In a previous report on spontaneous neoplasm in *Suncus murinus,* microtip-associated soft tissue sarcoma was found in the breeding colony of *Suncus murinus* [3].

The author has encountered spontaneous trichoblastomas derived from the facial skin with tactile hair in aged house musk shrews (*Suncus murinus*) of a breeding group. The aim of the current work was to report cases of trichoblastomas in tactile hair skin incidentally encountered in *Suncus murinus*. This paper describes the clinical findings, serum biochemical profiles (particularly, serum amyloid A levels) and histopathological results in *Suncus murinus*. This case report elucidate that the developmental and morphological characteristics in the tactile hair skin of *Suncus murinus* are different from those in humans, and cats and dogs.

## Case presentation

Male and female *Katmandu* strain *Suncus murinus* served as an outbred stock, which is a breeding group of genetically heterogeneous animals maintained as a closed colony without the introduction of animals from another stock. In the stock animals, we have incidentally encountered two cases (No. 1 and No. 2) of facial tumor involved in the pathogenesis of generalized wasting.

This *Suncus murinus* colony was primarily introduced at 9 weeks of age from Okayama University of Science. The animals were individually housed in cages (CL-0143 (R-2), W 355 × D 499 × H 198 mm, Crea Japan Inc., Tokyo, Japan) kept at a room temperature of 24 ± 2 °C, a relative humidity of 50 ± 5% and an air exchange rate of 15 times/hour. The room was artificially illuminated for 12 h/12 h. light–dark cycle daily (light 07:00–19:00, dark 19:00–07:00). The animals had free access to water bottles and a solid diet (d3, Feed One Co., Ltd., Yokahama, Japan).

Under genetic anesthesia with isoflurane, blood samples were collected from the caudal vena cava of the *Suncus murinus* using no anticoagulant. At 30 min after collection of blood samples, sera were separated by centrifugation at 1500 g for 10 min for biochemical analysis. For hematological samples, blood was collected into tubes containing K_2_EDTA.

In hematological examinations, the following parameters were examined using an automated cell counter (Microsemi LC-662 Horiba Co. Ltd, Kyoto, Japan): white blood cell count (WBC), red blood cell count (RBC), hemoglobin concentration (Hb), packed cell volume (PCV) ratio, mean corpuscular volume (MCV), mean corpuscular hemoglobin (MCH) and mean corpuscular hemoglobin concentration (MCHC).

In serum biochemical examinations, the following parameters were measured using a blood chemistry analyzer (Dry Chem NX 500 V: Fuji Film Co. Ltd, Tokyo, Japan): total protein (TP), albumin (ALB), albumin: globulin (A/G) ratio, total bilirubin (TBIL), blood urea nitrogen (BUN), creatinine (CRE), urate (UA), glucose (GLU), total cholesterol (TCHO), triglycerides (TG), asparate aminotransferase (AST), alanine aminotransferase (ALT), γ-glutamyl transpeptidase (GGT), lactate dehydrogenase (LDH), alkaline phosphatase (ALP), cholinesterase (ChE), leucine aminopeptidase (LAP), amylase (AMS), creatine kinase (CK), electrolytes (Na, K, Cl, Ca), inorganic phosphorus (IP) and magnesium (Mg).

In acute phase proteins, serum concentrations of serum amyloid A (vSAA) proteins were determined by using a latex agglutination turbidimetric immunoassay (SAA for animals, Eiken Chemical Co., Ltd., Tokyo, Japan, a trial reagent) and auto-chemistry analyzer method (HITACHI 7170S, Hitachi High-technologies Co., Ltd., Tokyo, Japan).

The suncus was euthanized by carbon dioxide exposure (a displacement rate 40% of the chamber volume/min). Immediately after euthanasia, the suncus was necropsied and tissue samples were taken for the histopathological examinations. The tissue specimens were fixed in 10% neutral buffered formalin, and 4-μm paraffin sections were stained with hematoxylin and eosine (HE), periodic acid/Schiff reaction (PAS), van Gieson’s stain and Weigert’s stain.

All procedures involving animals were approved by the Institutional Animal Care and Use Committee of Yamaguchi University and followed the Guidelines of Animal Care and Experiments of Yamaguchi University (approval No. 459). The animal care and use program for Advanced Research Center for Laboratory Animal Science in Yamaguchi University has been accredited by AAALAC International since 2018.

In the outbred colony, the author found a female and a male *Suncus murinus* suffering from the facial tumor and swelling with weight loss, malnutrition and coarse fur. The growth of the facial tumors gradually caused the difficulty in ingesting a diet, resulting in wasting in *Suncus murinus*. Both of the *Suncus murinus* presented with no metastasis to the other tissues and organs such as the liver, lungs and kidneys.

Hematological findings in the male animal are shown in Table 1. Although WBC moderately decreased, erythrocytic parameters (RBC, Hb and PCV ratio) apparently decreased as compared with the reference values. MCV, MCH and MCHC remined unchanged in the anemic status.

**Table 1:** Serum biochemical findings in the suncus suffering from mandibular tumors

Parameters	No. 1	No. 2	Reference values (mean ± SD)*	
Body weight (g)	41.0	61.0	Body weight range at sexual maturity Male: 50–70 Female: 30–50	

Sex	Female	Male		
Age (month)	14	16	The average of life span: 12–18	

Hematology			Male (13–20 M, n = 8)	Female (13–23 M, n = 7)
WBC (× 10^9^/L)	3.6	5.6	6.7 ± 1.9	6.8 ± 1.6
RBC (× 10^12^/L)	3.24	3.39	7.45 ± 0.39	7.96 ± 0.44
Hb (g/L)	57.0	55.0	151.7 ± 7.2	154.9 ± 8.5
PCV ratio	0.178	0.190	0.443 ± 0.019	0.487 ± 0.026
MCV (fL)	54.9	56.0	59.6 ± 1.6	61.1 ± 1.3
MCH (pg)	17.6	16.3	20.4 ± 1.2	19.5 ± 1.1
MCHC (g/L)	32.1	29.1	34.3 ± 1.1	31.9 ± 1.2

Serum biochemistry			Male (13–20 M, n = 8)	Female (13–23 M, n = 7)
TP (g/L)	56	65	55.6 ± 4.5	57.4 ± 9.2
Alb (g/L)	20	23	21.0 ± 7.7	23.9 ± 2.9
A/G ratio	0.56	0.55	0.74 ± 0.10	0.72 ± 0.09
T-BIL (μmol/L)	1.71	1.71	3.42 ± 2.42	2.69 ± 1.25
BUN (mmol/L)	32.6	43.8	24.91 ± 4.46	22.42 ± 3.47
CRE (μmol/L)	76.3	7.6	57.46 ± 26.89	36.62 ± 26.49
UA (μmol/L)	53.54	136.82	148.71 ± 62.81	68.83 ± 11.84
GLU (mmol/L)	5.35	13.68	12.53 ± 4.45	12.19 ± 5.58
T-CHO (mmol/L)	0.80	0.88	0.89 ± 0.13	1.04 ± 0.20
TG (mmol/L)	0.33	0.01	0.71 ± 0.54	0.29 ± 0.08
AST (U/L)	330	1000	609.38 ± 198.95	569.71 ± 231.04
ALT (U/L)	100	277	294.50 ± 141.03	210.57 ± 128.22
GGT (U/L)	5	9	29.63 ± 11.36	18.14 ± 14.99
LDH (U/L)	358	448	673.63 ± 237.64	530.57 ± 293.26
ALP (U/L)	78	92	59.25 ± 25.91	63.14 ± 11.03
ChE (U/L)	1	1	1 ± 0	1.43 ± 1.05
LAP (U/L)	29	53	38.38 ± 6.67	31.00 ± 4.57
AMY (U/L)	1388	1616	1338.75 ± 155.90	1386.14 ± 249.26
CK(U/L)	1941	2000	1502.13 ± 572.81	1044.86 ± 670.90
Na (mmol/L)	167	169	162.50 ± 2.45	161.14 ± 6.13
K (mmol/L)	4	6	4.56 ± 0.78	4.27 ± 0.57
Cl (mmol/L)	124	128	121.38 ± 3.60	121.86 ± 3.44
Ca (mmol/L)	2.48	2.25	2.69 ± 0.12	2.51 ± 0.14
IP (mmol/L)	3.91	4.85	2.97 ± 0.44	2.90 ± 0.58
Mg (mmol/L)	1.36	2.14	1.73 ± 0.19	1.67 ± 0.21
vSAA (μg/mL)	4.6	4.8	1.85 ± 1.10	2.01 ± 1.17

*Reference values in the laboratory of Laboratory Animal Science

Serum biochemical findings examined in the affected animals are shown in Table 1, in contradistinction to the author’s reference values in healthy *Suncus murinus* (n = 15 (male: 8, 13–20 months of age, female: 7, 13–23 months of age)). Overall, there were few apparent changes in the serum biochemical profiles in 2 *Suncus murinus*. The male *Suncus murinus* (Case No. 2) showed decreased TG concentrations and increased AST activities. Both the *Suncus murinus* exhibited the changes in electrolytes (decreased Ca and increased IP levels). vSAA used for a specific acute inflammatory marker was moderately high over the reference range.

**Fig. 1:**
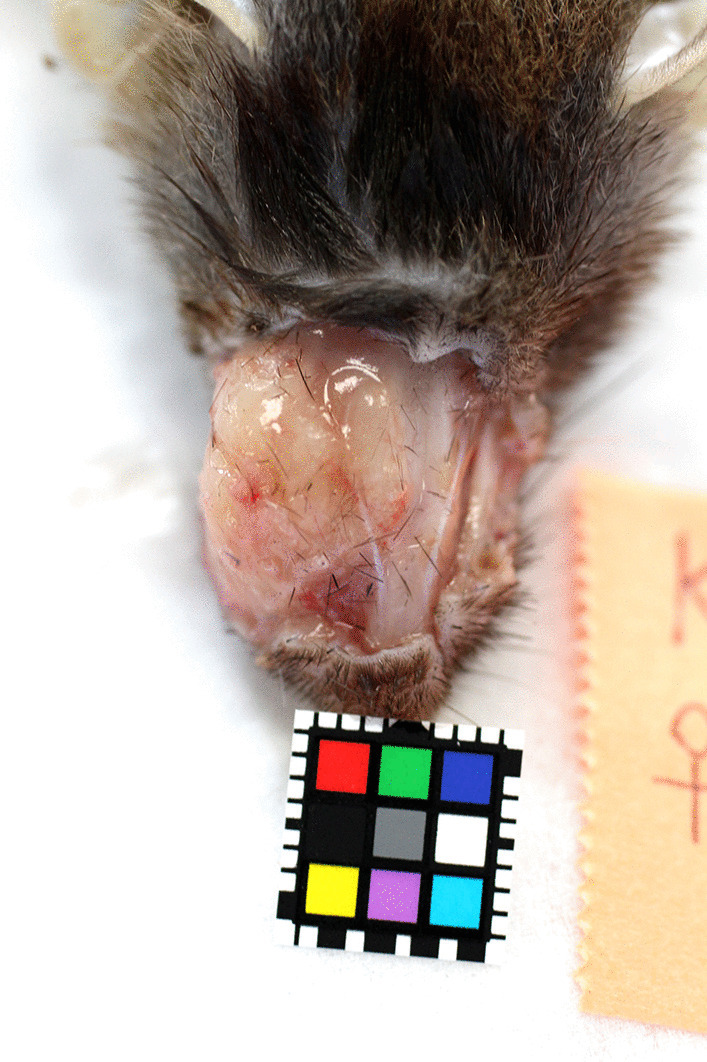
Macroscopic appearance of trichoblastoma in an aged Suncus murinus (Case No.1). An ovoid firm tumor is seen in the buccal area

Macroscopic appearance of the ovoid bulging lesion was shown in Fig. 1. A whitish firm tumor was solitarily observed in the right buccal area in Case No.1 and a similar tumor covered the right mandibula in Case No. 2. These mass lesions were not adherent to surrounding structures. Secondary changes such as ulceration, suppuration and hemorrhage were not accompanied by the tumor lesions.

**Fig. 2:**
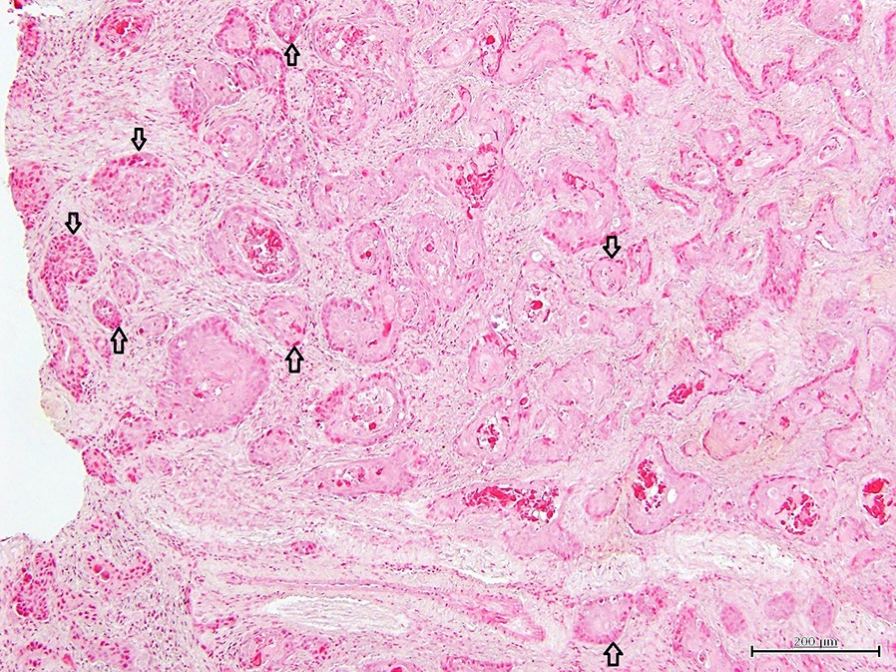
Microscopic finding of trichoblastoma (Case No.1). The nodule contains proliferation of follicular germ cells (open arrows). HE stain. × 100, Bar = 200 μm

**Fig. 3:**
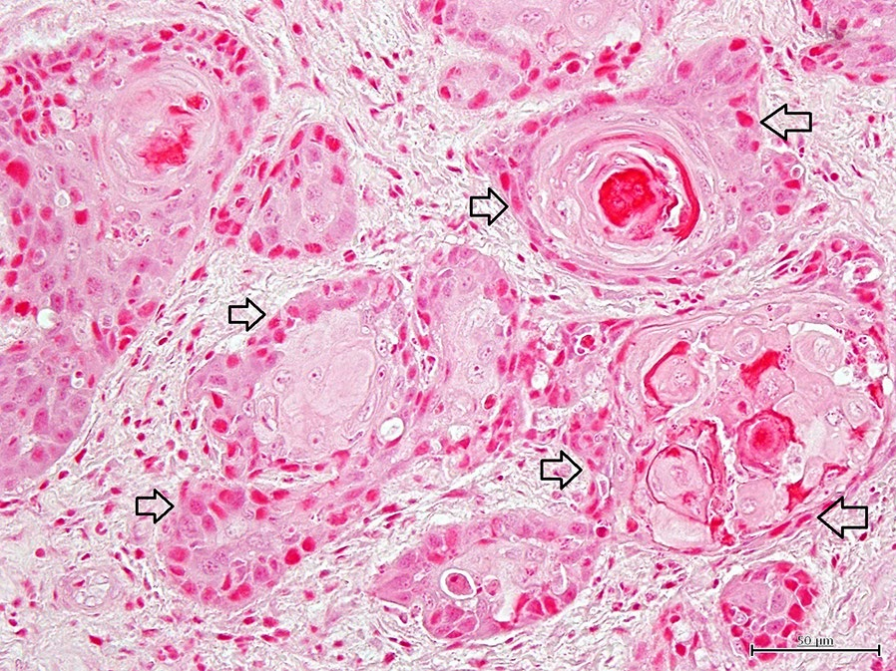
Microscopic finding of trichoblastoma (Case No.1). The cluster of basaloid tumor cells (open arrows) is seen. HE stain. × 400, Bar = 50 μm. Hair germ mesenchymal cells resemble follicular papillae

**Fig. 4:**
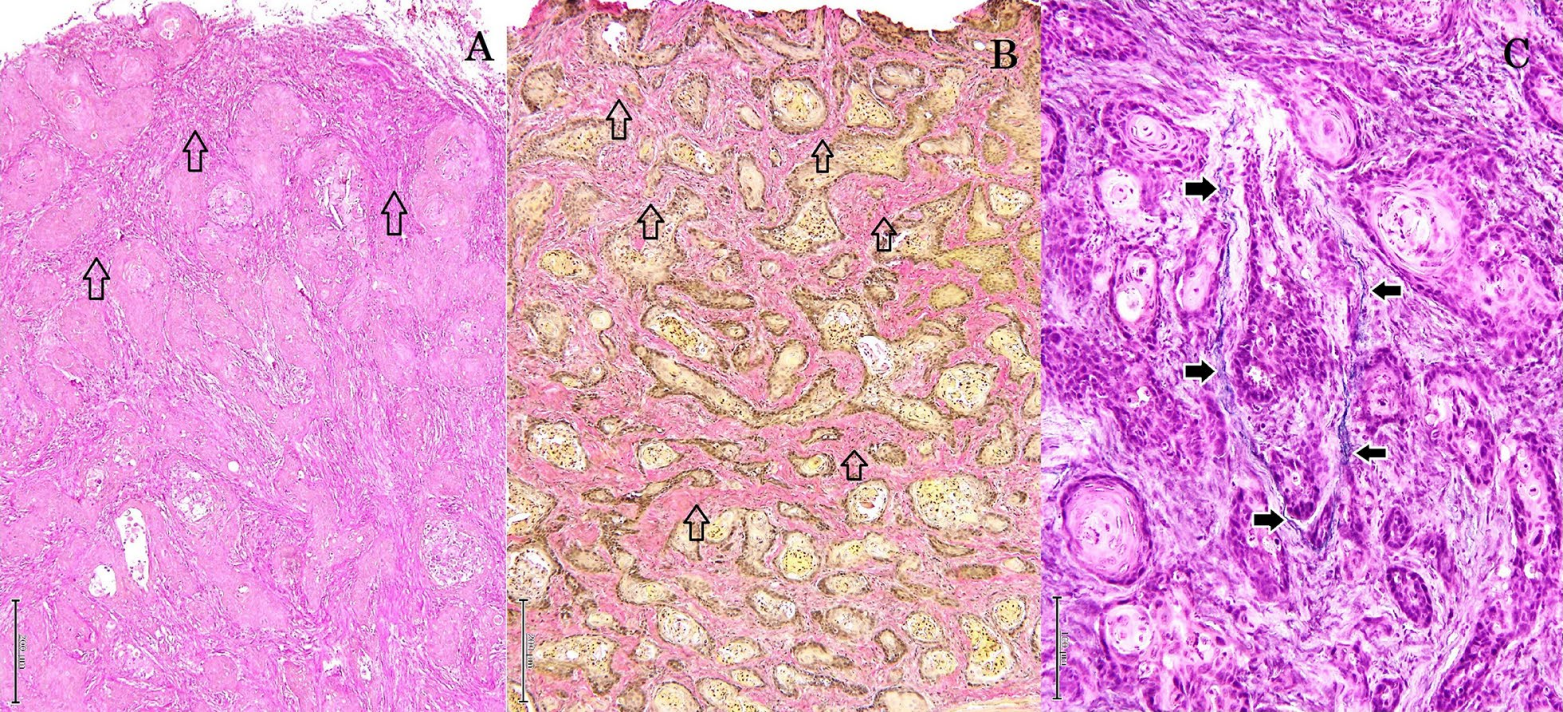
Microscopic finding of trichoblastoma (Case No.2). **A** PAS stain. × 100, Bar = 200 μm. The interstitial tissues contain PAS-positive collagen (open arrows) and elastic fibers. **B** van Gieson’s stain. × 100, Bar = 200 μm. Open arrows indicate collagen fibers. **C** Weigert’s stain. × 200, Bar = 100 μm. Solid arrows indicate elastic fibers

**Fig. 5:**
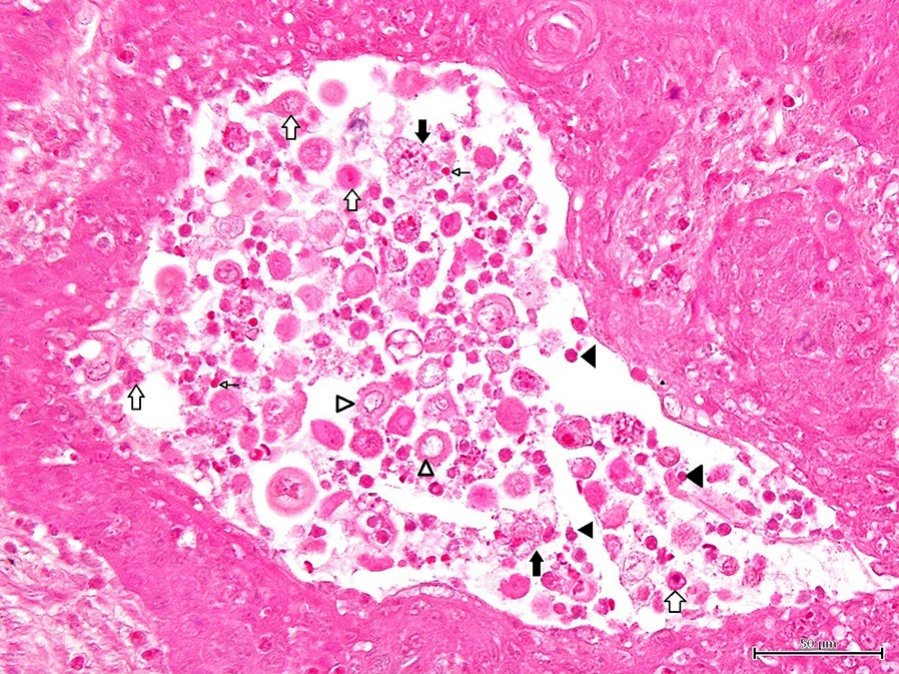
Microscopic finding of trichoblastoma (Case No.2). Microcysts contain various size of neoplasm cells (open arrows), giant cells (solid arrows), clear cells (open arrowheads), mononuclear cells (solid arrowheads) and erythrocytes (thin open arrows). HE stain. × 400, Bar = 50 μm

Histopathologically, the lesions consisted of abundant well-demarcated dermal nodules which were not located in proximity to the epidermis. The island structures revealed the presence of neoplastic cells with a cohesive and expansive growth pattern in dermal nodules. The nodules were composed of proliferation of follicular germ cells manifested by a combination of a variety of mesenchymal and epithelial cells (Fig. 2). Proliferation of epithelial cells was arranged in anastomosing cords. The cluster of basaloid tumor cells formed islands of hair germ mesenchymal cells resembling follicular papillae (Fig. 3). Follicular infundibulum or infundibular differentiation was not observed in the skin lesions, whereas follicular keratosis and cornifying follicular epithelium were founded in the dermis. There were also some small lacunae (cleft formation) between the interstitial tissues. The interstitial tissues contained collagen and elastic fibers (Fig. 4). In some portions, the island structures and dilated microcysts were arranged in the cord and trabeculae of basaloid keratinocytes. These microcysts included free floating various size of neoplasm cells, giant cells, clear cells, mononuclear cells and erythrocytes (Fig. 5). Degenerated keratinocyte and keratin fragments were not found in the cysts. In addition, neither sebaceous glands nor apocrine grands developed in the lesions. This section may be divided by subheadings. Although both of the cases showed similar histological findings, Case No. 2 had an apparent microcystic structure.

## Discussion and conclusions

In this case report, spontaneous trichoblastomas derived from the facial skin with tactile hair was serum biochemically and histopathologically examined in aged insectivorous *Suncus murinus*. To the best of author’s knowledge, this is the first report of trichoblastomas occurred in the tactile hair site in laboratory *Suncus murinus*. Although trichoblastomas are generally common in dogs and cats [2, 4–7], the neoplasms are uncommon or rare in human beings [8] and other species [9–14]. Trichoblastomas are relatively frequent and presents as a dermal or dermo-hypodermal nodule of the face, head and neck. The present cases also showed that the facial-cervical skin was a primary site of the occurrence of trichoblastomas in *Suncus murinus*.

Very little information is available concerning antemortem clinicopathological examinations in animals. In hematological examinations, the normal number of WBCs showed that *Suncus murinus* examined in this study had a low likelihood of infectious diseases. Erythrocytic parameters indicated that this animal was moderately affected with microcytic and normochromic anemia.

In serum biochemical examinations, there are few apparent changes in 2 *Suncus murinus* except for decreased TG concentrations and increased AST activities (Case No. 2). The increase in Ca and IP was probably influenced by age-related changes in the renal function. It was noteworthy that vSAA concentrations moderately increased during the chronic course of trichoblastomas. As reported in the author’s previous investigations [15, 16], vSAA agents proved useful in diagnosis of inflammatory disease in insectivorous species (Southern tamandua and *Suncus murinus*). Triggering events of neoplasia alter acute phase protein revels in animal species. Feline SAA increases with lymphoma and malignant mesothelioma and canine CRP increases with multicentric lymphoma, lymphatic neoplasia, hemangiosarcoma and mammary tumors. Acute phase response is a core part of the innate immune response [17, 18]. The present results observed in the primitive species such as *Suncus murinus* provide the following explanation for the role of SAA: SAA represents a conserved protein among mammals supporting the premise that it has a basic and essential role in the innate immune system. In addition, SAA shares a common mechanism feature across many animal species. Hematological and serum biochemical results provided the information on the benign tumor of trichoblastomas.

This tumor was histopathologically classified into several types, including ribbon, trabecular, granular cell and spindle cell [2]. The ribbon, medusoid and granular cell type is most often in dogs, while the spindle cell type is more frequent in cats. Trichoblastomas in human beings occur on head and are characterized by younger age, more frequency in women and longer duration [19]. In contrast, a retrospective study reported that the incidence of trichoblastomas were 2.0–2.6% in dogs and 2.0% in cats [20]. In dogs, most affected lesions are found in middle-aged and old animals (6–9 years of age). There is a predilection for the head and neck with the base of the ear being a typical site. In cats, although there is no known age or breed predilection, the tumors occur most often on the head and cranial half of the trunk [2]. In the present study, trichoblastomas were found in the facial area in aged *Suncus murinus*. The favorite sites in their trichoblastomas were similar to those in above-mentioned species.

Trichoblastomas in *Suncus murinus* showed the basaloid islands comprising peripheral palisading and a fibrocellular stoma. These histopasological findings accorded with those described for trichoblastomas in humans and other animals. This cutaneous tumor seemed to derives from differentiation of the hair germ of the developing follicles. The histopathological structures mimicked the hair bulbs and dermal hair papillae without the infundibula. The underdeveloped skin lesions were probably formed by recapitulation of the developing hair follicles. In a similar manner to human and rabbit trichoblastomas [13, 21], histopathological feature structurally resembled embryonic dermal condensation during hair follicle development.

Tactile hair is special hair that is induced by interstitial cells derived from neural crest cells. In dogs, cats and *Suncus murinus*, tactile hair possesses apparent hemarocele of hair follicles. The microcystic structure was characteristic of trichoblastoma occurred in the area with the tactile hair. Variable admixtures of free-floating cells suggested dermal inflammation and vSAA increases reflected this dermatopathological changes. The histopathological findings in this tactile hair site was different from those of trichoblastomas observed in other sites as well as in human trichoblastomas. It was probable that the hemarocele of hair follicles was associated with the dilated microcysts including various cell infiltration.

The author described the clinical findings, hematological and serum biochemical results, and histopathological characteristics of trichoblastomas in aged laboratory animal *Suncus murinus*. These lesions histopathologically showed the basaloid islands comprising peripheral palisading and dilated microcysts containing variable admixtures of free-floating cells. In conclusion, trichoblastomas in *Suncus murinus* revealed growth and morphological characteristics that recapitulate part of embryological development in the tactile hair follicles. In the histological structure, trichoblastomas in *Suncus murinus* were different from those observed in humans and animals such as cats, dogs and other wildlife.

## Data Availability

Data are available upon reasonable request to the corresponding author.

## Abbreviations

A/G: Albumin:globulin
ALB: Albumin
ALP: Alkaline phosphatase
ALT: Alanine aminotransferase
AMS: Amylase
AST: Asparate aminotransferase
BUN: Blood urea nitrogen
ChE: Cholinesterase
CK: Creatine kinase
CRE: Creatinine
GGT: γ-Glutamyl transpeptidase
GLU: Glucose
Hb: Hemoglobin concentration
HE: Hematoxylin and eosine
IP: Inorganic phosphorus
LAP: Leucine aminopeptidase
LDH: Lactate dehydrogenase
MCH: Mean corpuscular haemoglobin
MCHC: Mean corpuscular hemoglobin concentration
MCV: Mean corpuscular volume
PAS: Periodic acid/Schiff reaction
PCV: Packed cell volume
RBC: Red blood cell count
SAA: Serum amyloid A
TBIL: Total bilirubin
TCHO: Total cholesterol
TG: Triglycerides
TP: Total protein
UA: Urate
WBC: White blood cell count
